# Physical activity and ageing: The role of physiotherapy in promoting healthy ageing

**DOI:** 10.4102/sajp.v81i1.2114

**Published:** 2025-02-06

**Authors:** Shane Naidoo, Nirmala Naidoo

**Affiliations:** 1 Department of Health and Rehabilitation Sciences, Faculty of Health Sciences, University of Cape Town, Cape Town, South Africa

**Keywords:** aged, physical activity, non-communicable diseases, health ageing, cognition

## Abstract

**Background:**

The global rise in the older population, especially in sub-Saharan Africa, has heightened the impact of non-communicable diseases (NCDs), responsible for 74% of global deaths and the leading cause for years lived with disability. Physical activity (PA) has proven to manage NCDs; however, 80% of older adults in sub-Saharan Africa engage in low-to-moderate PA levels.

**Objectives:**

This literature review explores current evidence on the effects of PA on ageing and NCDs in older people in sub-Saharan Africa. Insights gained will enable physiotherapists to refine their PA prescriptions, aligning short-term rehabilitative goals with the broader scope NCD management while fostering principles of healthy ageing.

**Method:**

Electronic searches were conducted in: PubMed, EBSCOhost (Academic Search Premier – Africa-Wide Information, CINAHL, Health Sources Premier), Scopus and Google-Scholar to identify peer-reviewed studies published after 2010 related to PA, ageing, NCDs and older people (OP) in sub-Saharan Africa.

**Results:**

A structured PA protocol, comprising aerobic activity at 60% – 79% of maximal heart rate and resistance training at 50% – 60% of one-repetition maximum (3 weekly sessions each), proved effective in reducing NCDs. Integrating lifestyle behaviour changes further enhanced outcomes, notably improving blood sugar management and cardiac health.

**Conclusion:**

Structured aerobic and resistance PA, combined with lifestyle education, significantly reduces NCD risk factors in older adults, supporting healthy ageing.

**Clinical implications:**

The current research base in the field of ageing in SSA is limited, indicating the need for non-pharmacological interventions to manage the prevalence of NCDs, including in mental/cognitive health, where PA has a direct influence.

## Background

One of the greatest triumphs of the 21st century is the successful increase in human longevity. Global median life expectancy advanced from 64.5 years in 1990 to 71.5 years in 2017 (Stambler et al. 2018). In 2023, life expectancy averaged 75 years for men and 82 years for women in developed nations, while in the least developed nations, the averages were 63 and 67 years, respectively (Statista 2024). This improvement is predominately because of development in public health systems and amelioration of medical technology, which significantly decreased mortality that had previously resulted from communicable diseases. Academic predictions project that by 2050, the global population of older people (OP) will be 2.1 billion, of which 79% would be from developing countries (UN 2017). This phenomenon will see OP accounting for a larger percentage of the global population and is referred to as population ageing. Socioeconomic evolution is the major contributor to this projected statistic, as fertility rates have dropped contributing to fewer births and a smaller younger population. In Africa, high mortality rates arising from infectious diseases, maternal and/or infant mortality, violence and/or injury is contributing to a smaller population of younger people especially in sub-Saharan Africa (Naidoo, Otoo & Naidoo 2024).

South Africa (SA) has the highest and fastest accelerating population of OP on the African continent, from a reported 2.8 million in 1996 to a projected seven million by 2030 (Lehohla 2014). An increase in the OP population places health systems under strain as disorders in OP account for 23% of the global burden of disease (Smith et al. 2014). Older people have a high prevalence of multimorbidity, the most common being non-communicable diseases (NCDs), which increases the risk to contract viral diseases such as coronavirus disease 2019. Population ageing challenges countries’ resources because of decrease in labour force, higher fiscal demands with an increase in health expenditure and reduction in savings/investments of the working population. The financial and labour shortages could retard a country’s gross domestic product growth. Population ageing also impacts family dynamics (OP may require more social responsibility and care from the younger population) and the economy (retirees’ reliance on social grants from the state budget).

### Rationale

The prevalence of comorbidities and NCDs in OP globally, particularly in SA (45.3% hypertension, 15.8% diabetes, 13.3% arthritis), continues on its upwards trajectory (Murray et al. 2015). The SA study on ageing and global health showed that 81.7% of OP engaged in low-to-moderate levels of PA and the cohort were most at risk for developing NCDs than any other country. Physical activity has proven benefits in OP such as:

- decreasing the prevalence of NCDs (WHO 2018b)
- improve mental health and stagnate the onset of dementia (Lamb et al. 2018)
- increasing activities of daily living (Roberts et al. 2017)
- reducing the risk of falling (Gillespie et al. 2012)
- decreasing the incidence of stroke (Howard & McDonnell 2015)
- reducing the cost of future medical treatment (Towne et al. 2018).

Physical activity has been shown to increase independent living, decrease disability and improve quality of life. However, there is a dearth of literature regarding the influence of PA on the health and well-being of OP from this region. Our study will examine the outcomes of randomised controlled trials implementing PA interventions to manage or mitigate NCDs and their risk factors in OP residing in sub-Saharan Africa. Physiotherapists frequently prescribe structured PA programmes to support rehabilitation and to enhance/maintain functional capacity within this population. Their expertise in designing, implementing and educating on PA interventions positions them at the forefront of promoting health and well-being among OP. Insights gained from our study will empower physiotherapists to prescribe contextual programmes that extend beyond immediate rehabilitation goals, contributing to the broader aim of reducing NCD impact and promoting healthy ageing within this cohort.

### Ageing

**FIGURE 1:**
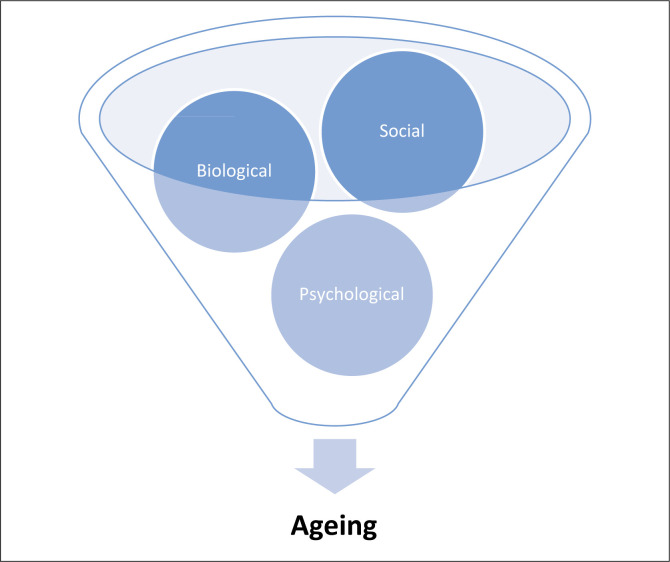
Ageing model in humans.

Ageing is a complex mechanism in humans that manifests on a biological, psychological and social level (Figure 1). The WHO defines biological ageing in humans as the impact of a wide variety of cellular and molecular changes that occur over time. These changes contribute to cells becoming less efficient, losing their ability to divide and repair themselves and are referred to as cellular senescence. In healthy individuals, senescent cells can self-destruct by a process of apoptosis or are removed by the immune system to maintain homeostasis. As the age of an individual increases, these homeostatic regulators lose their functional ability, which leads to the accumulation of senescent cells in tissue and organs. The build-up of senescent cells leads to decline in physiological function and age-related NCDs. The rate at which biological ageing occurs is dependent on a combination of extrinsic (smoking, unhealthy diet, pollution) and intrinsic (genetically predetermined) factors (Koopman et al. 2015). Psychological ageing often entails neurobiological changes, such as reduced white matter integrity and cerebral blood flow, which can impair memory, learning and emotional responsiveness (Schaie & Willis 2010). Social ageing refers to the changes in the relationship an individual has with family, friends and people in an organisation they involved in (work, prayer group), as their chronological age increases. These changes are often interlinked and the rate of the cumulative effect and the stage that they occur in an individual’s lifespan differs.

### Physical activity

Physical activity is defined as any movement produced by the skeletal muscles that require the expenditure of energy (WHO 2018a). The WHO recommends age-specific PA guidelines to maintain satisfactory levels of health; however, 80% of adolescents and 27% of adults globally do not meet these levels (WHO 2018a). Physical inactivity has been proven to have direct correlations with the prevalence of NCDs and listed as one of its risk factors (smoking, alcohol abuse, unhealthy diet and air pollution being the others) (WHO 2016). Globally, physical inactivity has been reported to be the fundamental cause of 25% breast/colon cancer, 27% diabetes and 30% ischaemic heart disease (Cunningham et al. 2020). Non-communicable diseases account for 41 million global mortalities annually, of which 77% are from low- and middle-income countries. In SA, mortality resulting from NCDs saw a rise of 58.6% between 1997 and 2018 with a median age of 65 years and 69 years for males and females, respectively (Statssa 2018). Ageing is a risk factor for multimorbidity, with a global prevalence of 51.0%, and reports of between 30 and 87% in OP from SA (Roomaney et al. 2021).

### Biological ageing

#### Cardiovascular system

Ageing is a risk factor for vascular diseases, the most prevalent being stiffening and buildup of plaque in vessels of the heart referred to as atherosclerosis, which is often the precursor to most cardiovascular diseases (CVD). Senescent cells have been found in plaque lining the vessels of the heart indicating that biological ageing is a risk factor for atherosclerosis. Obesity, poor nutrition, physical inactivity and smoking have been listed as modifiable risk factors for CVD, with obesity being the major modifiable risk factor to effect change in CVD such as type 2 diabetes, hypertension and dyslipidaemia. Results from a systematic review showed that individuals who were physically active had considerably lower arterial stiffness compared to peers who were sedentary (Park et al. 2017). Diabetes, cerebrovascular diseases and hypertension disorders were the top three leading causes of death in OP in SA in 2018, accounting for 26.1% of deaths in this demographic (Statssa 2018). Cardiovascular diseases are the most common cause of death in people with diabetes, which is compounded by the occurrence of hypertension.

A study in Nigeria investigated the effect of a continuous PA intervention over 8 weeks (3 × week; weeks 1–2, 45 min cycling 60% – 79% maximum heart rate (HR); weeks 3–8, 60 min cycling 79% maximum HR) that produced decreases in systolic (−13.94 ± 6.95 mmHg) and diastolic (−7.41 ± 6.26mmHg) blood pressure (Sikiru & Okoye 2014). These results concurred with a study implementing a continuous PA programme over 8 weeks that displayed decrease in oxygen saturation (± 3.9%), resting HR (±4.8 beats/min), systolic (±15.0 mmHg) and diastolic (±7.9 mmHg) blood pressure. An independent investigation examined the impact of continuous and interval PA programmes on blood pressure, revealing significant reductions in both systolic and diastolic measurements (Lamina 2010). Nonetheless, comparisons between the interval and continuous PA groups disclosed no significant differences in these parameters. This could indicate that aerobic PA conducted between 60% and 79% maximum HR, either continuous or interval, is effective in the management of blood pressure in this cohort. A randomised controlled trial studied the effects of an aerobic and strengthening PA programme (3 × 60 min/week over 12 weeks) that demonstrated a decrease in resting HR, resting respiratory rate with increases in oxygen saturation, however, had no significant change in blood pressure (Ajiboye et al. 2013).

Two independent studies that trialled an intervention of educational PA and lifestyle modification (healthy diet, reducing alcohol consumption and cessation of smoking) produced no change in blood pressure (Fairall et al. 2016; Goudge et al. 2018). These results concur with a study conducted in SA implementing a similar intervention that produced no significant changes in diastolic and systolic blood pressures (Steyn et al. 2013). However, a study on lifestyle modification and education on a structured PA programme produced significant reduction in systolic (−4.06 mmHg) and diastolic (−2.4 mmHg) blood pressure (Okube, Kimani & Mirie 2022). These results could indicate that PA interventions should be structured to be effective in this cohort.

#### Musculoskeletal system

There is involuntary loss of muscle mass (3% – 8%) and strength from the third decade of life, which is accelerated from the sixth decade, referred to as sarcopenia (Volpi, Nazemi & Fujita 2004). Sarcopenia is a complex age-related condition that generally results from changes in muscle structure, hormonal imbalances and metabolic variations, resulting in muscle atrophy (Petermann-Rocha et al. 2022). It affects around 10% of OP globally and is linked with unfavourable health conditions such as falls, fractures and disability (Shafiee et al. 2017). Obesity and physical inactivity are compounding factors to sarcopenia and vice versa. Sarcopenia is one of the leading attributes driving functional decline and loss of independence in OP and is interlinked with frailty (Dodds & Sayer 2016).

An investigation into the effect of aerobic PA on OP with sarcopenia demonstrated improvements in physical performance, however, no change in muscle mass (Liu et al. 2014). A study carried out on OP compared the effect of a nutrition supplement combined with an aerobic component on muscle mass and strength and showed that adding a nutritional supplement to a PA programme improved muscle mass (Gualano et al. 2014). These results concurred with an empirical study that reported adding a nutritional supplement to a PA intervention produced superior results in muscle and strength gains (Atherton et al. 2020). A randomised controlled trial conducted among OP in sub-Saharan Africa implemented a PA intervention incorporating resistance PA, resulting in a significant improvement in muscle composition outcomes (Ajiboye et al. 2013).

Osteoporosis, often termed the silent disease (as it frequently manifests only when a fracture occurs), is the most common skeletal condition that affects OP with a global prevalence of 18.3%, with Africa boasting the unenviable statistic of the highest global prevalence of 39.5% (Salari et al. 2021). Osteoporosis is a systemic bone disorder that is identified by progressive loss in bone density, with degeneration of structure and tissue. This increases the risk of fragility fractures, which impacts morbidity and mortality. It is prevalent in post-menopausal women and approximated that 40% of post-menopausal females and 30% males are prone to osteoporotic fractures (Sözen, Özişik & Başaran 2017). Autophagy dysfunction, which is a feature of ageing, has been linked to bone disorders such as osteoporosis. Risk factors for osteoporotic fractures include family history of hip fractures, use of oral corticosteroids, blood disorders such as multiple myeloma and modifiable risk factors such as smoking, frequent alcohol consumption and physical inactivity. Decrease in skeletal height has been linked to age-related bone density changes, which is further compounded by the presence of sarcopenia. A meta-analysis on the effect of PA on bone mineral density reported that participants in the PA group had higher bone mineral density scores in the lumbar spine and the femoral neck post-intervention (Martyn-St James & Carroll 2009). These results diverge from those of an independent study, which found that although PA had no significant impact on bone mineral density, it positively affected balance, strength and fall reduction, all critical fracture risk factors (Pinheiro et al. 2020). A scholarly analysis reported a 29% reduction in fractures when comparing the highest with the lowest levels of PA (Qu et al. 2014). Since 2010, no interventional research on PA has been undertaken in sub-Saharan Africa to evaluate its effects on bone density in osteoporosis. The high costs associated with the necessary imaging could be a contributing factor.

Osteoarthritis is the most common age-related joint disorder that affects 80% of OP with a global prevalence of 7%, accounting for 2% of the global years lived with disability (Hunter, March & Chew 2020). It is the most ubiquitous form of arthritis in SA affecting 29.5% urban and up to 82.7% rural residing OP (Rangiah, Govender & Badat 2020). Regardless, it is one of the most overlooked NCD notwithstanding its compounding effects on disability, especially when occurring concurrently with comorbidities. Age has been listed as the most pronounced risk factor for osteoarthritis because of cellular senescence buildup and autophagy dysfunction leading to joint and cartilage degeneration (Loeser 2011). Obesity is the most modifiable risk factor for osteoarthritis and has been shown to have direct correlations with its prevalence, particularly in the knee (Zheng & Chen 2015). Physical activity has been shown to have an indirect correlation with obesity and is one of the leading non-pharmaceutical/non-surgical interventions for the management of osteoarthritis (Daste et al. 2021).

Lower back pain is predicted to affect 843 million globally by 2050, with ageing being one of the driving factors. Global prevalence of lower back pain in OP varies from 21% – 75%, with Nigeria reporting a prevalence of 38.5% in rural and 41.5% in urban areas (De Souza et al. 2019). The majority of back and/or neck pain in OP is non-specific and can arise from pathological and/or physiological or psychosomatic conditions. Results from a systematic review on the benefits of PA interventions on OP with lower back pain reported that PA can reduce pain and disability (Vadalà et al. 2020). Furthermore, OP with chronic lower back pain show trends of sedentary behaviour, aggravating the negative clinical ramifications of ageing.

### Body composition

There are approximately 2.3 billion overweight and 700 million obese older adults globally. In SA, the prevalence of obesity among individuals with osteoporosis is 46.1%, with 49.1% exhibiting elevated waist circumference measurements (Romano et al. 2021). The authors further reported that there was a positive correlation with obesity and 5 of the 12 chronic conditions analysed (hypertension, arthritis, chronic back pain, asthma and diabetes). This concurs with a study that recorded a high prevalence of overweight and obesity in OP from SA (Phaswana-Mafuya et al. 2013). Obesity has been reported to mirror the effects of ageing (telomere shortening, mitochondrial anomalies, debilitated immunity, inadequate autophagy and increased inflammation), indicating that an obese OP is more likely to have comorbidities and functional impairments than a healthier peer (Amarya, Singh & Sabharwal 2018). In contrast, it has been reported that a BMI between 31 kg/m^2^–35 kg/m^2^ and 28 kg/m^2^–31 kg/m^2^ was linked to a lower mortality rate than a BMI < 25 kg/m^2^ in OP > 70 years of age (McKee & John 2021). The theory postulates that this cohort benefits from the positive effects of having excess fat deposits (prevention of malnutrition, safeguarding from excess bone mineral loss and cognitive declines); as this cohort has successfully navigated the harmful effects of obesity and survived. Age-related changes in body composition may not have an influence on body weight or BMI as increases in body fat may be negated by loss in lean mass. Ageing has shown to have a direct correlation with visceral fat accumulation and an indirect correlation with sub-cutaneous fat in the body. An increase in visceral fat is strongly associated with unfavourable health conditions such as CVD, type II diabetes and cancer (Chang et al. 2012). Changes in body composition associated with ageing might compromise the reliability of anthropometric measures such as waist circumference, BMI and waist-to-hip ratio. These metrics may not accurately reflect the distribution of fat mass in OP, potentially reflecting misleading clinical assessments.

High-intensity PA (75% VO_2_ max) has positive results in decreasing visceral fat in OP as compared to medium-intensity PA (50% VO_2_ max) (Coker et al. 2009). An empirical study reported that there is a 63% less chance of an OP to develop metabolic syndrome if they engaged in high levels of PA (150+ min moderate-to-vigorous PA) (Xu et al. 2022). These results agree with a high-intensity aerobic and resistive PA intervention in Nigeria (12 weeks, 3 sessions/week 60 min), which reported significant changes for BMI (Ajiboye et al. 2013). A clinical trial documented substantial BMI reduction following an 8-week interval training regimen (3 × 60-min sessions per week) at 60% – 79% of maximum HR (Lamina et al. 2014). In a study conducted on OP in SA, a Pilates intervention was trialled over 8 weeks (3 sessions/week 60 min) at a low intensity and reported no significant changes in body mass and BMI (Fourie et al. 2013). These results indicate that the dosage and intensity of PA are essential to effect significant changes in body fat in OP.

An educational lifestyle intervention study conducted in Rwanda over 12 months (45–60-min sessions/week on diet, regular PA, cessation of smoking, alcohol abuse and adherence to medication) produced favourable results for body weight (Amendezo et al. 2017). An independent clinical trial also trialled an educational lifestyle intervention over 12 months, however, it had no significant changes to body weight or BMI (Owolabi et al. 2019). A similar lifestyle intervention study conducted in SA also showed no significant changes in BMI and waist circumference, however, showed a significant decrease in systolic (−4.65 mmHg) and diastolic (−3.30 mmHg) blood pressure (Mash et al. 2014). However, a peer-led educational lifestyle management intervention conducted over 1 year produced significant decreases in BMI and waist circumference (Debussche et al. 2018). An empirical study that trialled a continuous PA intervention over 8 weeks (3 × week, 60 min 79% max HR) produced significant decreases in waist-hip-ratio (−0.08 cm), BMI (−0.07 kg/m^2^) and percentage body fat (−1.01%) (Sikiru & Okoye 2014). An aerobic and resistive PA intervention produced significant decreases in percentage body fat and fat mass (Ajiboye et al. 2013). A Pilates intervention conducted over 8 weeks (3 sessions/week 60 min) showed no significant changes in body mass and BMI (Fourie et al. 2013). This could indicate that PA interventions should have an aerobic and/or a resistive component to have significant decreases in BMI.

### Nervous system

The nervous system is composed of the central (brain and spinal cord) and peripheral (spinal and cranial nerves) nervous system. Changes in the structure of the brainstem that occur in biological ageing could have a negative effect on the functions of the autonomic nervous system. Cerebral atrophy is one of the profound effects ageing can have on the nervous system, as much as 5% per decade, after an individual enters the fourth decade of life. Cerebral atrophy can result in aphasia, dementia and in extreme cases seizures. Mortality is known to be higher in OPs who have disorders of dementia, which is further compounded by comorbidities. There are approximately 50 million people living with dementia globally, which is expected to surpass 152.8 million by 2050. There was an estimated 2.13 million people in SSA living with dementia in 2015; however, this number is predicted to rise by 257% by 2050 (Guerchet et al. 2017).

Physical activity has shown to have positive effects on the health of the brain and on the general psychological well-being of an individual. High-intensity PA was associated with a 28% reduction in dementia risk, while moderate-intensity PA was credited with lowering the risk by 24% among OP (Guure et al. 2017). An intervention study carried out in Nigeria on individuals diagnosed with dementia, used progressive task-orientated circuit training, which demonstrated positive clinical outcomes in cognition, physical functioning and societal participation (Gbiri & Amusa 2020). An unconventional intervention of backward gait training on participants with Parkinson’s disease demonstrated minimal cognitive improvement (Grobbelaar, Venter & Welman 2017). A novelty intervention study of sports cup stacking had no significant effect on balance or cognition (Naidoo & Moodley 2018).

Ageing in the peripheral nervous system influences axonal atrophy and degeneration of the myelin sheath. Ageing has an indirect correlation with the peripheral nervous system regeneration properties following deterioration or injury. Axonal health can be further compromised in OPs who have comorbidities (diabetes, vascular diseases). These effects compromise the electrophysiologic properties of the peripheral nerves and influence sensation, nerve conduction speed as well as autonomic responses. Age-related changes in the autonomic system could lead to decreased automatic regulation of blood pressure and uncoordinated autonomic conduct (e.g. bladder control). Sensorimotor peripheral nerve function declines with older age and leads to decline in muscle strength, power and bone density especially in the lower extremities, consequently increasing the risk of falls in this cohort. The global prevalence of peripheral neuropathy is 2.4%, elevated to 8% in OP (Hammi & Yeung 2019). Results from a 12-week, moderate-intensity PA programme implemented on OP with peripheral neuropathy showed positive clinical results in nerve conductivity and neuropathic symptoms (Gholami et al. 2021). These results concur with results from a study reporting that moderate-intensity PA conducted at least 5 minimum sessions per week of 30 min was beneficial for individuals with autonomic dysfunction (Fu & Levine 2013). A PA intervention of aerobic dance in OP showed results of improved autonomic cardiac control, emphasising the importance of PA in this cohort (Varas-Diaz et al. 2020).

## Psychological and social ageing

The biological effects of ageing on the nervous system potentially lead to deterioration of cognitive function; however, ageing also has profound effects on the emotional response to various life and social situations. Two out of every five OP in sub-Saharan Africa experience symptoms of depression, with a prevalence of 40% in SA (Adamek & Kotecho 2023). Anxiety and compromised physical well-being are reported to be linked to death/fear-of-death to a significant person and fear-of-death is related to depression and low self-esteem. Physical activity is inversely related to depression, with interventions yielding significant clinical improvements in depressive symptoms within this population. These results are in agreement with a study in Tunisia on OP, which trialled a PA resistance and aerobic intervention over 4 months (4 × week: 60 min) with a significant reduction in anxiety and depression scores (Frih et al. 2017).

Loneliness is prevalent in ageing and some of the risk factors are (Somes 2021) as follows:

- death of loved one/spouse
- a lack of involvement with family (when children leave home)
- change of environment (e.g., relocation to an old age facility)
- retirement – a lack of social interaction with work colleagues
- social stigma associated with ageing disorders (e.g., bladder/bowel incontinence) could lead to social embarrassment and increase the risk isolation
- decreased financial resources after retirement to facilitate socialisation.

Loneliness has been shown to increase the risk of cardiovascular diseases mortality by 90%, dementia by 50% and stroke by 32% in OP (Somes 2021). Physical activity programmes have shown to decrease the effects of loneliness in OP. A physical activity intervention programme implemented in Nigeria in OP over 12 weeks produced significant increases in societal participation, physical functioning and cognition (Gbiri & Amusa 2020).

## Summary

Evidence indicates that adherence to a rigorously structured PA protocol, incorporating aerobic PA at 60% – 79% of maximal heart rate (three weekly sessions of 45–60 min) combined with resistance training starting at 50% of one-repetition maximum (three weekly sessions of 3 sets of 10–12 repetitions per muscle group, progressively increasing to 60%), demonstrated optimal efficacy in reducing NCDs and/or their associated risk factors within this cohort when sustained for a minimum of 8 weeks. However, when we strategically combine this with targeted education on lifestyle behaviours (healthy diet, cessation of smoking and reduced alcohol consumption), we unlock even greater benefits, especially in critical areas such as blood sugar management and cardiac health.

The beneficial effects of PA on health and well-being underscore its potential to enhance the health and quality of life of OP in sub-Saharan Africa, with outcomes that include reducing chronic disease risk, improving mental health and elevating overall quality of life. There is sufficient evidence to support the need for culturally sensitive interventions that consider the unique social and environmental factors influencing physical activity among OP in SSA. Physiotherapists are instrumental in designing and promoting targeted PA interventions for OP, leveraging their expertise in functional assessment and rehabilitation to prescribe PA regimens that enhance physical capacity, counteract age-related decline and foster healthy aging. This uniquely positions them to support overall health, longevity and quality of life in the ageing population.
